# Quasi-Situ Characterization of Retained Austenite Orientation in Quenching and Partitioning Steel via Uniaxial Tensile Tests

**DOI:** 10.3390/ma13204609

**Published:** 2020-10-16

**Authors:** Pengfei Gao, Jie Liu, Weijian Chen, Feng Li, Jingyu Pang, Zhengzhi Zhao

**Affiliations:** 1 Collaborative Innovation Center of Steel Technology, University of Science and Technology Beijing, Beijing 100083, China; b20160476@xs.ustb.edu.cn (P.G.); liujie@ustb.edu.cn (J.L.); b20180510@xs.ustb.edu.cn (W.C.); b20160477@xs.ustb.edu.cn (F.L.); g20189182@xs.ustb.edu.cn (J.P.); 2 Beijing Laboratory of Metallic Materials and Processing for Modern Transportation, University of Science and Technology Beijing, Beijing 100083, China; 3 Beijing Engineering Technology Research Center of Special Steel for Traffic and Energy, University of Science and Technology Beijing, Beijing 100083, China

**Keywords:** microstructure, mechanical properties, anisotropy, quenching and partitioning steel, retained austenite, quasi-situ electron backscatter diffraction

## Abstract

As a representative of the third generation of advanced high strength steel, the quenching and partitioning steel has excellent potential in automobile manufacturing. The characterization and analysis of the mechanical properties and microstructure of the quenching and partitioning steel during deformation is an effective way to explore the microstructure evolution and transformation-induced plasticity effects of complex phase steels. The relationship between the microstructure morphology and mechanical properties of a 1180 MPa-grade quenching and partitioning steel was investigated through interrupted uniaxial tensile tests plus quasi-situ electron backscatter diffraction measurements. A mixture of ferrite, martensite, and retained austenite was observed in the microstructure. It was found that the volume fraction of global retained austenite decreased linearly with the increase of displacement (0 mm–1.05 mm). The evolution of the retained austenite with typical crystal direction ranges with deformation was characterized. Results show that the orientation (111) and (311) account for the highest proportion of retained austenite grains in the undeformed sample and the mechanical stability of the (311) retained austenite grains is the best. Moreover, the retained austenite grains rotated significantly in the early stage of the specimen deformation process (around yielding), and the work hardening of the specimen was weak at this stage, simultaneously.

## 1. Introduction

Steel has been important material for body construction of motor vehicles in North America since the early 1900s. Light weight represents a trend for the modern automotive industry with the demands of energy conservation and emission reduction. In order to achieve lightweight vehicles, development of the advanced high strength steel (AHSS) is required. Up till now, researches for the high-strength steel mainly focus on generating high-strength matrix structure and ensuring enough retained austenite content [1,2]. As a third generation of advanced high-strength steel proposed in 2003 [3], quenching and partitioning (Q&P) steel can meet the requirements of high strength and high plasticity at the same time. Through control of heat treatment, Q&P steel can obtain low C (carbon) ferrite, low C martensite, and high C retained austenite at room temperature. Studies have shown that the metastable austenite, which can be present at room temperature, could transform to martensite during straining, greatly enhancing the strength and ductility [4,5].

Stability of retained austenite has always been the focus of research on Q&P steel and other advanced high-strength steels. The influence factors in previous researches are summarized as follows: the stability of retained austenite improves with the increase of C content, while decreasing with the grain size due to the low martensitic transformation temperature; the stability of thin film-like retained austenite is higher than that of block and other shapes. In addition, the retained austenite stability is also related to texture [6,7,8,9].

However, it is limited to study the microstructure evolution during deformation under conventional quasi-static deformation. By using quasi-situ electron backscatter diffraction (EBSD) measurements [10,11,12] to study the evolution of the retained austenite (RA) fraction with deformation, we can clearly characterize the morphology and volume fraction evolution of RA grains with typical orientation. In the present work, 1180 MPa-grade Q&P steel was used as the investigated material. Multiple techniques, including X-ray diffraction (XRD), scanning electron microscopy (SEM), and transmission electron microscopy (TEM), were used for observation and characterization. The evolution of microstructure with strain was studied through interrupted tensile tests plus quasi-situ EBSD measurements.

## 2. Materials and Methods

**Figure 1:**
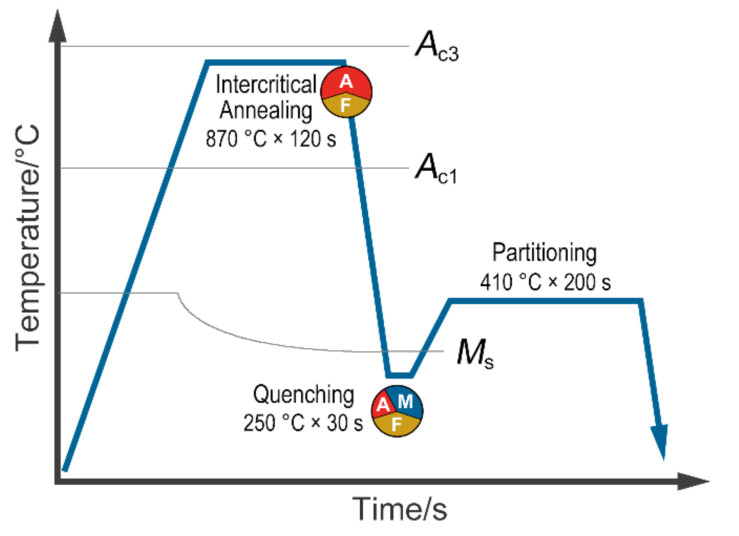
The heat treatment process diagram.

A 1180 MPa-grade Q&P steel was investigated in the present study. Table 1 shows the main chemical composition (in mass fraction) of the investigated material. Carbon and manganese were added to stabilize austenite [13], and silicon was used to inhibit the formation of cementite [14]. The investigated steel was produced by a two-step Q&P process [15]. Figure 1 shows the heat treatment process and parameters. The ingot was prepared by vacuum induction melting and then forged to 40 mm. The forge piece was reheated at 1200 °C for 2 h, hot-rolled to 2.8 mm, followed by simulated coiling at 660 °C. After cold-rolling to 1.6 mm, the steel sheet was intercritically austenitized (870 °C × 120 s) between *A*_c1_ (austenite transformation starting temperature) and *A*_c3_ (austenite transformation finishing temperature), followed by quenching to temperatures (250 °C × 30 s) between the *M*_s_ (martensite transformation starting temperature) and *M*_f_ (martensite transformation finishing temperature), then reheated and isothermally held above *M*_s_ (410 °C × 200 s) before final quenching to room temperature.

The microstructures were characterized by SEM and TEM. A Quanta 450 FEG field emission scanning electron microscope (FEI, Hillsboro, OR, USA) (operated at 20 kV) and a JEM 2100 transmission electron microscope (JEOL Ltd., Tokyo, Japan) (operated at 200 kV) were used to characterize the microstructure of the investigated steel. Metallographic samples were first cut by wire electrical-discharge machining, then mechanical ground and polished, finally etched with 3% (in volume fraction) nital. The thin foil samples for TEM observation were prepared by twin-jet polishing and ion beam thinning.

XRD test was carried out on a Bruker D8 Advance diffractometer (Bruker, Karlsruhe, Germany). The XRD test was performed using LynxEye XE^TM^ detector (Bruker, Karlsruhe, Germany) and Cu*Kα* radiation operating at 40 kV and 40 mA. The scan mode was set as continuous power spectral density (PSD) fast, and the diffraction angle parameter (2 Theta) was set to 47–94°, with an increment of 0.02°. The amount of RA was calculated by the following equation [16].

(1) $$V_{\gamma }=1{.4I}_{\gamma }/\left(I_{\alpha }+1.4I_{\gamma }\right)$$

where *V*_γ_ is the volume fraction of retained austenite, *I_γ_* and *I_α_* are the average integral intensities of the FCC (face-centered-cubic) and BCC (body-centered-cubic) peaks. Diffraction peaks BCC-(200), BCC-(211), FCC-(200), FCC-(220), and FCC-(311) are considered.

**Figure 2:**
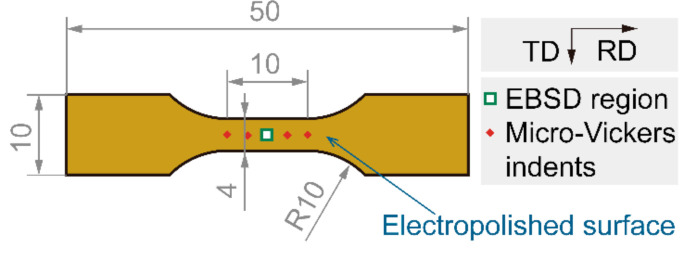
Schematic diagrams of interrupted uniaxial tensile plus quasi-situ EBSD samples. Dimensions in mm, TD: transverse direction, RD: rolling direction, the same below.

Tensile samples with a gauge length of 10 mm and a gauge width of 4 mm, as shown in Figure 2, were used for interrupted uniaxial tensile plus quasi-situ EBSD experiment [17] and quasi-tensile test. The samples were wire-cut from the heat-treated sheets, and the long axis was kept paralleling to the RD (rolling direction) of the sheet. The tensile sample’s surface was prepared by electropolishing (10% perchloric acid alcohol, 10 V, ~1 A). Interrupted uniaxial tensile test and quasi-tensile test were carried out at room temperature on a CMT5605 tensile tester (SANS, Shanghai, China) with a rate of about 3 × 10^−4^ s^−1^. To investigate the local microstructure evolution with the increasing tensile strain, quasi-situ EBSD experiments were carried out by a PHI710 auger electron spectrometer (ULVAC-PHI, Inc., Chigasaki, Japan) with an EDAX EBSD probe (EDAX, Mahwah, NJ, USA) (with a working voltage of 20 kV and a step size of 60 nm). The EBSD data were acquired from the same region with the guidance of micro-Vickers indents and were analyzed using orientation imaging microscopy (OIM) software (Version: 7.3.0 x86, EDAX, Mahwah, NJ, USA).

## 3. Results and Discussion

### 3.1. Microstructures and Quantitative Metallography Analysis

**Figure 3:**
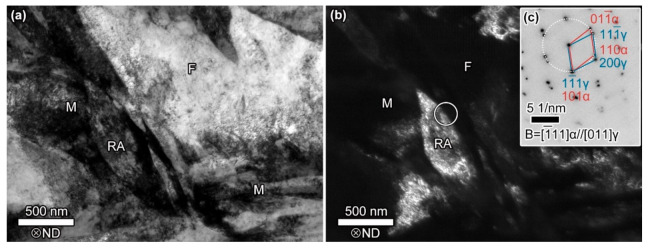
TEM analyses of the investigated steel. (**a**) bright-field (BF) image, (**b**) dark-field (DF) image of RA corresponding to (**a**) with (**c**) selected electron diffraction pattern of circled area in (**b**). The F, RA, and M denote the ferrite, retained austenite, and martensite, respectively. ND: normal direction, the same below.

**Figure 4:**
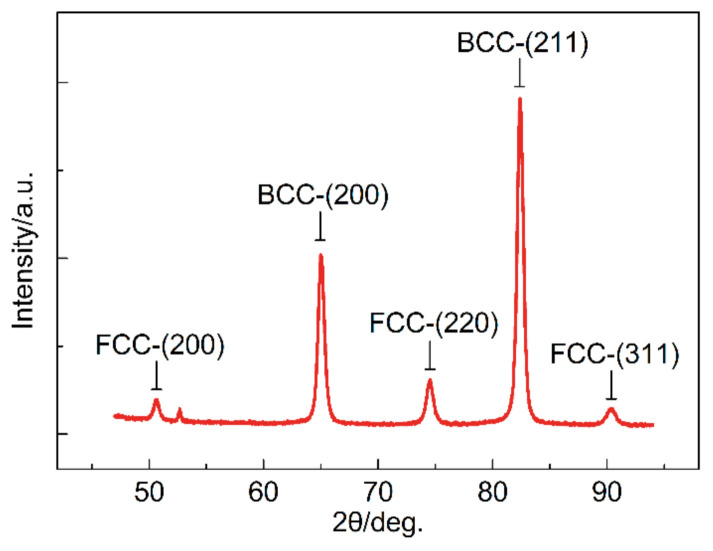
X-ray diffraction peak pattern of the investigated steel.

**Figure 5:**
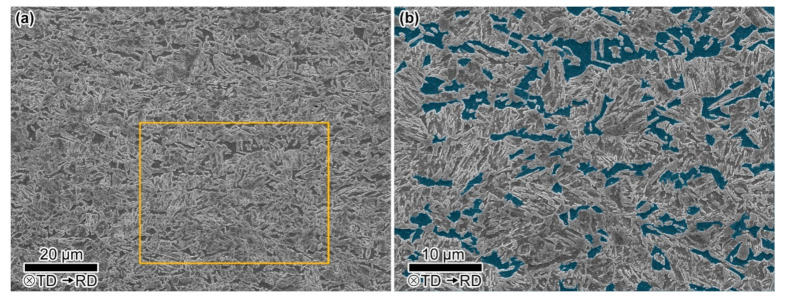
SEM microstructures of the investigated steel. (**b**) shows the microstructure details within the yellow box in (**a**) and the ferrite grains are superimposed in blue.

The TEM photos of the investigated steel are shown in Figure 3, from which ferrite, martensite and RA can be identified clearly. The ferrite in the investigated steel is formed during the intercritical isothermal treatment, and its volume fraction is related to the annealing temperature. The volume fraction of RA is related to quenching and partitioning process. The Q&P steel needs enough RA content to ensure the TRIP (Transformation-Induced Plasticity) effect and improve mechanical properties. According to the analysis of XRD data (Figure 4), the volume fraction of RA is 11.4%. The microstructure of the investigated steel in a larger field of view is characterized by SEM (Figure 5). We used quantitative metallographic technology to identify and label ferrite, and the rest is a mixture of martensite and RA. No secondary martensite was observed in the investigated steel.

Table 2 lists the phase volume fraction of the investigated steel, in which the volume fraction of ferrite is calculated from 10 SEM photos, the volume fraction of RA is obtained from XRD data, and then the rest is the volume fraction of martensite. Realizing the composite effect [18] and TRIP (transformation induced plasticity) effect is the purpose of the microstructure design of the intercritical annealed Q&P steel. The composite effect is a feature of multiphase steel. The soft and hard phases in the multiphase steel can benefit plasticity and strength, respectively, through partitioning of micro-stress during strain. Compared with the first proposed completely austenitized Q&P process, the intercritical annealed process introduces soft phase ferrite into the Q&P steel and refines the grains by setting a lower annealing temperature. Simultaneously, the soft/hard phase ratio can be adjusted by settling the isothermal temperature of intercritical annealing. A relatively high isothermal temperature is selected for higher strength, and only 18.1% ferrite was obtained in the investigated steel. The essential feature of the TRIP effect is considered as a significant stress redistribution between FCC and BCC. Sufficient (11.4% in the investigated steel) RA ensures the continuous occurrence of TRIP effects during deformation.

### 3.2. Macroscopic Stress-Displacement Responses

**Figure 6:**
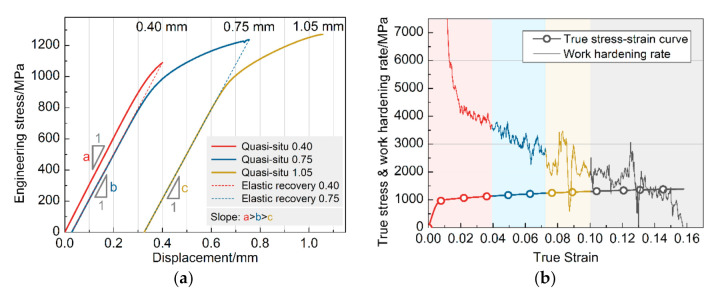
Tensile curves of the investigated steel. (**a**) Engineering stress-displacement curves obtained during interrupted uniaxial tensile plus quasi-situ EBSD experiment. (**b**) True stress-strain curves and work hardening rates obtained during quasi static tensile test. The red, blue and yellow marks on the curve correspond to different displacements (0.4, 0.75, 1.05 mm) of interrupted uniaxial tension respectively.

To characterize the structural evolution of microstructure during plastic deformation, the interrupted tensile tests plus quasi-situ EBSD measurements were performed on the investigated steel. Figure 6a shows the engineering stress-displacement curves obtained from the interrupted tensile tests. After three times of quasi-static stress application, the tensile specimen obtained a total elongation of ~10.5% in the axial direction. The maximum stress reached in the three tensile stages is 1088 MPa, 1235 MPa, and 1270 MPa, and continuous yielding is observed in each stage.

As shown in Figure 6a, three solid lines record the stress-displacement relationship measured by the test equipment during the loading process. The two dashed straight lines represent the sample’s elastic recovery when unloaded, which is determined during data analysis. The slope of the previous stage of unloading is equal to the next stage of the loading slope. The slope of the three stages’ elastic stage decreases gradually, which is caused by the deformation-induced martensite transformation. The elastic modulus of multiphase material is related to each phase’s elastic modulus and the volume fraction. During the interrupted tensile, the volume fraction of RA decreases, and the volume fraction of martensite increases due to deformation-induced martensite transformation. Simultaneously, the elastic modulus of martensite is smaller than that of austenite, leading to a decrease in slope, that is, a > b > c (a = 3.06 GPa/mm, b = 2.93 GPa/mm, c = 2.88 GPa/mm).

Figure 6b shows the true stress-strain curves and work hardening rates obtained during the quasi-static tensile test. The continuous jitter phenomenon exists in all stages of the overall work hardening rate curve, demonstrating the sustained TRIP effect of RA, and contributes to the high elongation of the investigated steel [19]. It is worth noting that, compared to the early stage of deformation (around yielding), this kind of jitter is more significant in the middle and late stages of deformation, which means a more extensive TRIP effect.

### 3.3. Evolution of the Global RA Fraction with Deformation

**Figure 7:**
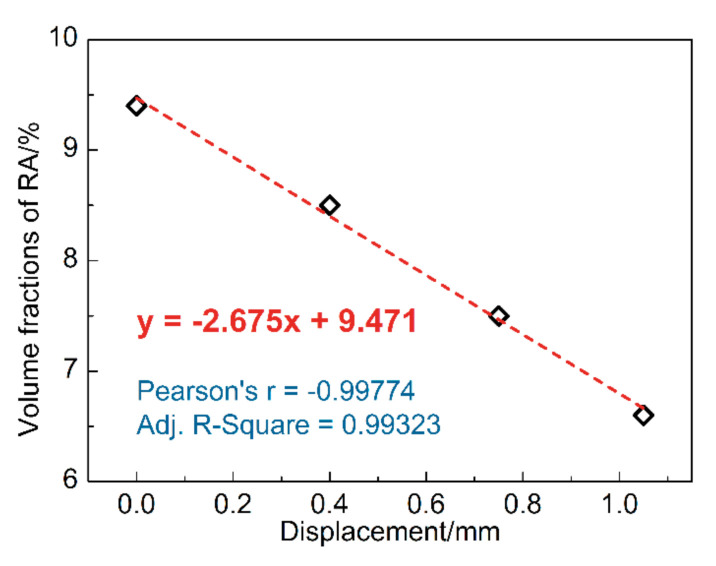
Evolution of the global RA fraction with deformation.

We determine the evolution of global RA fraction with increasing deformation via EBSD in the same area during interrupted uniaxial tensile. Approximately considering the deformation within the gauge length of the specimen, displacement can represent the change of strain to a certain extent. As shown in Figure 7, the relationship can be expressed as a linear function. The intercept 9.471 is associated with the starting austenite fraction. Further, the slope −2.675 is considered to be related to the stability of RA, which is decided by the carbon content, grain morphology, orientation, etc. of RA.

It is noticed that the RA fraction of the undeformed sample (9.4%) seemed to be less than that measured by XRD (11.4%). Due to the change in the microscopic stress state between the grains during the grinding and polishing process, part of the RA undergoes deformation-induced martensitic transformation [20]. However, as a bulk technique, the XRD test can avoid this error to a certain extent. Therefore, we introduce XRD to analyze the proportion of each phase of the undeformed specimen (Table 2) and use EBSD data to qualitatively analyze the sample’s microstructure evolution during the deformation process.

### 3.4. Evolution of the RA with Typical Orientation

**Figure 8:**
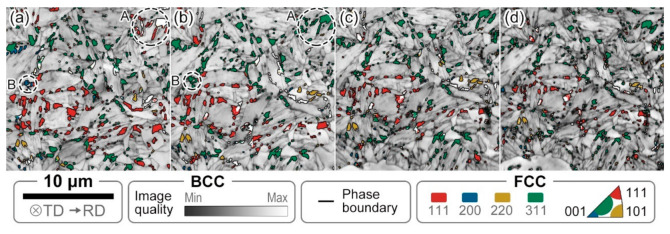
Combined quasi-situ typical textures distribution maps of RA and image quality (IQ) maps at different displacements of (**a**) 0 mm; (**b**) 0.40 mm; (**c**) 0.75 mm and (**d**) 1.05 mm.

**Figure 9:**
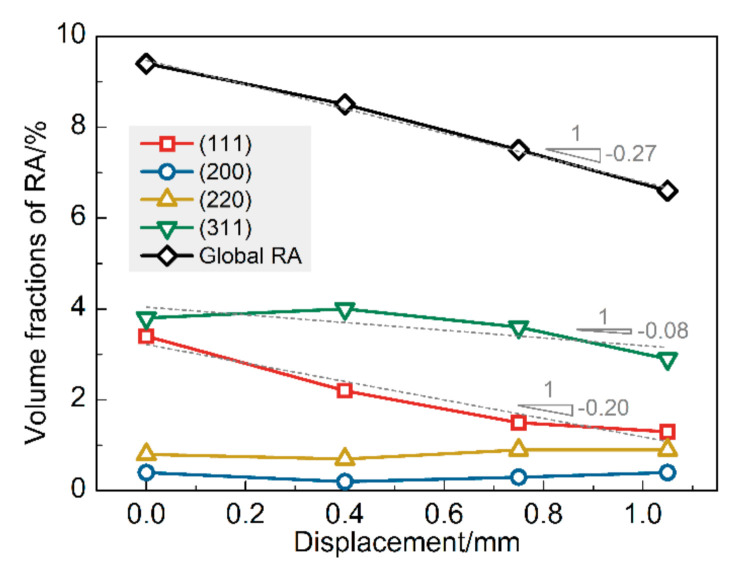
The volume fraction of typical orientation RA grain with varying displacements.

Figure 8 shows the evolution of microstructure with deformation (average confidence index > 0.26; average image quality > 89,674.85; average fit < 1.56°). The gray scale represents the image quality (IQ) of the BCC phase (ferrite and martensite), and the color indicates the typical orientation (Misorientation angle: 0°–15°) distribution of RA. Furthermore, the RA volume fraction evolution of each texture is shown in Figure 9. The figures give us a clear recognition that FCC’s main crystal direction in the undeformed sheet is (311) and (111), which respectively accounts for 3.8% and 3.4% volume fraction of the microstructure. The volume fraction of (200) and (220) Ra grains is relatively low (<1%) and remains constant during the deformation process.

The dotted lines in Figure 9 are the linear fit of the volume fraction of RA with deformation, representing the decay trend. The slope of the linear fit of (311) RA is smaller than that of (111), that is, (311) RA grains have higher mechanical stability. Figure 8 shows that the deformation results in a rotation of the RA grain, especially in the initial deformation stage, around the displacement of 0.4 mm. The crystal direction ranges of the RA grains identified by dashed circles A and B have been rotated from (111) and (200) to (311), respectively. That also leads to the (311) RA curve in Figure 9 peaks at 0.4 mm displacement, resulting from the superposition of deformation-induced martensite transformation and RA grain rotation. The rotation of the RA grains is considered to improve the RA grains’ stability and thus improve the plasticity of the materials [21,22]. Analyzing the factors affecting the plasticity of the test steel, the rotation of RA mainly occurs in the early stage of deformation, and with the increase of deformation, the TRIP effect gradually increases.

**Figure 10:**
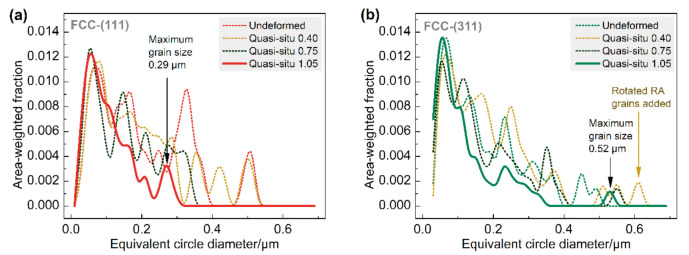
The size distribution of (**a**) (111) and (**b**) (311) orientation RA grains under different displacements.

Figure 10 characterizes the difference between (111) and (311) crystal direction ranges from the evolution of RA grain size distribution with deformation. The area-weighted algorithm is used to calculate the particle size distribution, which can result in a better-looking distribution of the grain size, without affecting any of the statistics. The RA grain distribution curves of the two typical orientations all move to the left with the increase of deformation, which characterizes the partial phase transition of RA grains. The maximum RA grain size of the (111) and (311) orientations is about 0.5 μm in the undeformed sample. After 1.05 mm displacement of the sample, the maximum RA particle size of (111) orientation is reduced to 0.29 μm, while that of (311) is still 0.52 μm, which reflects the higher stability of (311) orientation RA grains.

Orientation dependence of martensitic transformation prevails strongly during dynamic transformation under deformation. That selectivity of phase-transformation to orientation can be ascribed to the influence of martensite variants’ mechanical work in different orientation RA grains [23]. Compared with (111) RA, which is more prone to deformation-induced martensitic transformation, the formation of the BCC phase is suppressed in (220) and (200) RA during tension. The non-phase-transformed RA grains act as a soft phase in the deformation process. Compared with (111) RA, this kind of RA has a weaker contribution to the overall plasticity and work hardening of the material. The higher volume fraction of (111) RA and the lower volume fraction of (220) and (200) RA in the investigated steel ensure the occurrence of the TRIP effect, which is beneficial to the overall mechanical properties.

## 4. Conclusions

In this paper, the microstructure deformation behavior of the investigated Q&P steel is investigated through interrupted tensile tests plus quasi-situ EBSD measurements. The evolution of the RA with a typical crystal direction ranges with deformation is characterized. The study also characterizes and reveals the contribution of RA grain rotation to the high mechanical stability of (311) oriented RA grains.

1. The volume fraction of global RA decreases linearly with the increase of deformation. When the displacement increases from 0 mm to 1.05 mm, the volume fraction of global RA decreases from 9.4% to 6.6% according to EBSD data.
2. The (111) and (311) grains account for the highest proportion of RA in the undeformed sample (3.8% and 3.4%, respectively). Simultaneously, the latter has higher mechanical stability when the material is deformed.
3. It was observed from quasi-situ typical textures distribution maps of RA that the deformation resulted in the rotation of the RA grain. Meanwhile, the RA grains are more inclined to (311) orientation with higher mechanical stability. At the same time, the work hardening rate of the material remains low at the strain stage when the RA grains rotation occurs significantly.

## Figures and Tables

**Table 1:** Chemical composition of investigated steel (wt.%).

C	Si	Mn	Fe
0.18–0.22	1.50–2.0	2.10–2.70	Balance

**Table 2:** Phase volume fraction of the investigated steel (vol.%).

Retained Austenite	Ferrite	Martensite
11.4	18.1	70.5

## References

[1] Jacques P., Furnémont Q., Lani F., Pardoen T., Delannay F. (2007). Multiscale mechanics of TRIP-assisted multiphase steels: I. Characterization and mechanical testing. Acta Mater..

[2] Wang Y., Geng H., Zhu B., Wang Z., Zhang Y. (2018). Carbon Redistribution and Microstructural Evolution Study during Two-Stage Quenching and Partitioning Process of High-Strength Steels by Modeling. Materials.

[3] Speer J., Streicher A., Matlock D., Rizzo F., Krauss G. (2003). Quenching and partitioning: A fundamentally new process to create high strength trip sheet microstructures. Austenite Form. Decompos. Proc. Symp..

[4] Liu C., Peng Q., Xue Z., Wang S., Yang C. (2018). Microstructure and Mechanical Properties of Hot- Rolled and Cold-Rolled Medium-Mn TRIP Steels. Materials.

[5] Chen Y., Wang H., Cai H., Li J., Chen Y. (2018). Role of Reversed Austenite Behavior in Determining Microstructure and Toughness of Advanced Medium Mn Steel by Welding Thermal Cycle. Materials.

[6] Basuki A., Aernoudt E. (1999). Influence of rolling of TRIP steel in the intercritical region on the stability of retained austenite. J. Mater. Process. Technol..

[7] Jimenez-Melero E., van Dijk N., Zhao L., Sietsma J., Offerman S., Wright J., van der Zwaag S. (2007). Martensitic transformation of individual grains in low-alloyed TRIP steels. Scr. Mater..

[8] Song C., Yu H., Li L., Zhou T., Lu J., Liu X. (2016). The stability of retained austenite at different locations during straining of I&Q&P steel. Mater. Sci. Eng. A.

[9] Wang C., Chang Y., Yang J., Cao W., Dong H., Wang Y. (2016). Work hardening behavior and stability of retained austenite for quenched and partitioned steels. J. Iron Steel Res. Int..

[10] Ding R., Dai Z., Huang M., Yang Z., Zhang C., Chen H. (2018). Effect of pre-existed austenite on austenite reversion and mechanical behavior of an Fe-0.2 C-8Mn-2Al medium Mn steel. Acta Mater..

[11] Long X., Zhang R., Zhang F., Du G., Zhao X. (2019). Study on quasi-in-situ tensile deformation behavior in medium-carbon carbide-free bainitic steel. Mater. Sci. Eng. A.

[12] Zhao J., Zhang F. (2020). In-situ observation of tensile deformation and retained austenite transformation behaviors in carbide-free bainitic steel. Mater. Sci. Eng. A.

[13] De Moor E., Lacroix S., Clarke A.J., Penning J., Speer J. (2008). Effect of retained austenite stabilized via quench and partitioning on the strain hardening of martensitic steels. Metall. Mater. Trans. A.

[14] Devaraj A., Xu Z., Abu-Farha F., Sun X., Hector L. (2018). Nanoscale solute partitioning and carbide precipitation in a multiphase trip steel analyzed by atom probe tomography. JOM.

[15] Coryell J., Savic V., Hector J., Mishra S. (2013). Temperature effects on the deformation and fracture of a quenched-and-partitioned steel. SAE Tech. Pap..

[16] Tanaka M., Choi C. (1972). The Effects of Carbon Contents and M_s_ Temperatures on the Hardness of Martensitic Fe-Ni-C Alloys. Trans. Iron Steel Inst. Jpn..

[17] Gao P., Chen W., Li F., Ning B., Zhao Z. (2020). New crystallography insights of retained austenite transformation in an intercritical annealed quenching and partitioning steel. Mater. Lett..

[18] Fu B., Yang W., Wang Y., Li L., Sun Z., Ren Y. (2014). Micromechanical behavior of TRIP-assisted multiphase steels studied with in situ high-energy X-ray diffraction. Acta Mater..

[19] Liang J., Zhao Z., Tang D., Ye N., Yang S., Liu W. (2018). Improved microstructural homogeneity and mechanical property of medium manganese steel with mn segregation banding by alternating lath matrix. Mater. Sci. Eng. A.

[20] Tsuchiyama T., Tobata J., Tao T., Nakada N., Takaki S. (2012). Quenching and partitioning treatment of a low-carbon martensitic stainless steel. Mater. Sci. Eng. A.

[21] Li W., Gao H., Nakashima H., Hata S., Tian W. (2016). In-situ EBSD study of deformation behavior of retained austenite in a low-carbon quenching and partitioning steel via uniaxial tensile tests. Mater. Charact..

[22] Knijf D., Foejer C., Kestens L., Petrov R. (2015). Factors influencing the austenite stability during tensile testing of Quenching and Partitioning steel determined via in-situ Electron Backscatter Diffraction. Mater. Sci. Eng. A.

[23] Ma D., Yang P., Gu X., Onuki Y., Sato S. (2020). In-situ neutron diffraction investigation on the martensite transformation, texture evolution and martensite reversion in high manganese TRIP steel. Mater. Charact..

